# Measurement of Liver Blood Flow: A Review

**DOI:** 10.1155/1991/68915

**Published:** 1991

**Authors:** A. M. Seifalian, G. P. Stansby, K. E. F. Hobbs, D. J. Hawkes, A. C. F. Colchester

**Affiliations:** 1 Department of Hepatobiliary Surgery and Liver Transplantation Royal Free Hospital School of Medicine Pond Street London NW3 2QG UK; 2 Division of Radiological Sciences United Medical and Dental School Guy's Hospital Campus London SE1 9RT UK; 3 Department of Neurology United Medical and Dental School Guy's Hospital Campus London SE1 9RT UK

## Abstract

The study of hepatic haemodynamics is of importance in understanding both hepatic physiology and disease processes as well as assessing the effects of portosystemic shunting and liver transplantation. The liver has the most complicated circulation of any organ and many physiological and pathological processes can affect it^1,2^. This review surveys the methods available for assessing liver blood flow, examines the different parameters being measured and outlines problems of applicability and interpretation for each technique.

The classification of these techniques is to some extent arbitrary and several so called “different” methods may share certain common principles. The methods reviewed have been classified into two groups (Table 1): those primarily reflecting flow through discrete vessels or to the whole organ and those used to assess local microcirculatory blood flow. All techniques have their advantages and disadvantages and in some situations a combination may provide the most information. In addition, because of the many factors affecting liver blood flow and sinusoidal perfusion, readings in a single subject may vary depending on positioning, recent food intake, anxiety, anaesthesia and drug therapy. This must be borne in mind if different studies are to be meaningfully compared.


*HPB Surgery*, 1991, Vol. 4, pp. 171–186
Reprints available directly from the publisher
Photocopying permitted by license only

© 1991 Harwood Academic Publishers GmbH
Printed in the United Kingdom

### REVIEW ARTICLE

### MEASUREMENT OF LIVER BLOOD FLOW: A REVIEW

A.M. SEIFALIAN, G.P. STANSBY and K.E.F. HOBBS
*Department of Hepatobiliary Surgery and Liver Transplantation, Royal Free Hospital School of Medicine, London NW3 2QG, UK*

D.J. HAWKES
*Division of Radiological Sciences, United Medical and Dental School, Guy's Hospital Campus, London SE1 9RT, UK*

A.C.F. COLCHESTER
*Department of Neurology, United Medical and Dental School, Guy's Hospital Campus, London SE1 9RT, UK*

*(Received 7 February 1991)*

The study of hepatic haemodynamics is of importance in understanding both hepatic physiology and disease processes as well as assessing the effects of portosystemic shunting and liver transplantation. The liver has the most complicated circulation of any organ and many physiological and pathological processes can affect it[1,2]. This review surveys the methods available for assessing liver blood flow, examines the different parameters being measured and outlines problems of applicability and interpretation for each technique.

The classification of these techniques is to some extent arbitrary and several so called "different" methods may share certain common principles. The methods reviewed have been classified into two groups (Table 1): those primarily reflecting flow through discrete vessels or to the whole organ and those used to assess local microcirculatory blood flow. All techniques have their advantages and disadvantages and in some situations a combination may provide the most information. In addition, because of the many factors affecting liver blood flow and sinusoidal perfusion, readings in a single subject may vary depending on positioning, recent food intake, anxiety, anaesthesia and drug therapy. This must be borne in mind if different studies are to be meaningfully compared.

KEY WORDS: Blood flow, tissue perfusion, portal blood flow, hepatic blood flow, Doppler ultrasound, Doppler laser, x-ray angiography

Address correspondence to: Mr. Alexander M. Seifalian, Department of Hepatobiliary Surgery and Liver Transplantation, Royal Free Hospital School of Medicine, Pond Street, London, NW3 2QG, UK.

**Table 1** Hepatic blood flow measurement techniques.

| |
|---|
| METHODS MEASURING BLOOD FLOW |
| *Velocity or Transit Time Methods* |
| Electromagnetic Flowmeter |
| Dopler Ultrasound |
| X-Ray Angiography |
| Nuclear Magnetic Resonance |
| *Dye Dilution Techniques* |
| Plasma Disappearance Method |
| Radioisotope techniques |
| METHODS MEASURING TISSUE PERFUSION |
| Radiolabelled Microspheres |
| Heat Exchange |
| Hydrogen Electrode |
| Oxygen Electrode |
| Laser Doppler |

### METHODS MEASURING BLOOD FLOW

### *Velocity or Transit Time Methods*

#### *Electromagnetic flowmeter*

The measurement of blood flow by electromagnetic induction was first suggested by Fabre[3] and the principle of the technique is based on Faraday's law of electromagnetic induction. If a magnetic field is applied across a vessel in which blood is flowing then an electric field is induced at right angles both to the induced magnetic field and the flow vector[4,5]. The electrical field is detected along its axis from the potential difference across the outside of the vessel. This potential is primarily determined by the velocity of the flowing blood within the vessel. Accuracy demands attention to detail and proper calibration[6,7] using a pump and saline solution. There is no way of checking calibration *in vivo* except vessel clamping for zero flow. Interference from other electrical instruments also minimise the accuracy of the technique[8].

In practice the method involves the placement of the device around the vessel to be assessed. For a good signal, close contact is essential. Drapanas, *et al*[9] and Price *et al.*[10] compared electromagnetic flowmetry with the bromsulphalein clearance method[11] for measuring hepatic arterial and portal vein flow in the dog and found a good correlation between the two methods. Because of the invasive nature of the technique, it is more applicable to animal studies and on patients at the time of surgery. These devices are, however, still regarded as the "gold standard" against which all other methods of measuring flow must be compared. They are able to measure instantaneous and mean blood flow in an exposed vessel. They can detect forward and reverse flow and the temporal resolution is fast enough for flow to be studied during the cardiac cycle. Other advantages of the method are its insensitivity to changes in blood temperature and viscosity.

*Doppler ultrasound*

The first attempted use of Doppler ultrasound for the measurement of blood flow from the surface of the body was reported by Satomura in 1959[12] but compared to ultrasound imaging the role of Doppler ultrasound has evolved slowly and has largely been restricted to a relatively few well-defined indications in cardiac diagnosis, evaluation of carotid and peripheral vascular disease, and more recently in obstetrics and the abdomen[13,14]. The combination of real time B-mode ultrasound imaging and a pulsed Doppler flowmeter is referred to as a duplex scanner[15]. Using these machines the diameter of the vessel, peak velocity, mean velocity, volume flow rate, and pulsatility of blood flow waveforms can be measured[16]. A recent refinement is the development of colour flow mapping where the image provides flow information concerning all structures in the image field rather than just at one selected site.

Duplex ultrasound offers the best non-invasive way of assessing portal vein patency[17] and can demonstrate cavernous transformation and whether portal flow is hepatopetal or hepatofugal. Doppler has also been used in quantitative measurement of portal blood flow. Ohnishi *et al.*[18] compared a pulsed Doppler flowmeter with cineangiography[19] for calculating portal vein velocity in normal volunteers and patients with liver disease. Doppler and cineangiographic measurements exhibited significant correlation ($r = 0.960$; $n = 31$; $p < 0.001$) for velocities from 2.4 to 12.2 cm/sec, but Doppler values were consistently about twice that of the cineangiographic values, underlying the need for calibration against a flow model. They suggested that Doppler might be of more use for assessing relative changes in portal flow rather than for giving absolute flow values. Ackroyd *et al.*[20] stated that raw flow values vary from person to person according to body weight, state of fasting, and position, as well as anxiety level and exercise and suggested that the use of standardised conditions could improve accuracy. One further reason for not relying on portal velocity measurements in studying cirrhotic patients is that portal flow is well maintained by portal vein dilatation until portal hypertension is severe[21]. For this reason a portal congestion index, which is the cross sectional area divided by the mean velocity, has been suggested by Moriyasu *et al.*[22] as being more useful than absolute flow values.

The Duplex scanner can also be a useful non-invasive tool for assessing patients with portosystemic shunts. Nelson *et al.*[23] studied patients before and after portosystemic shunting with both duplex scanning and angiography. They concluded Duplex was accurate in determining the direction of flow if an adequate tracing was obtained. Preoperatively it allows the determination of portal vein patency and direction of flow. Postoperatively most porto-caval and mesocaval shunts can be visualised as well as some Warren type shunts. As with many ultrasound applications success is largely dependent on operator skill and experience. Patency is directly demonstrated by flow in the correct direction and a confirmatory sign is the demonstration of corresponding phasic patterns of blood flow in the portal vein and inferior vena cava. Other confirmatory signs are dilatation of the inferior vena cava proximal to portocaval and mesocaval shunts and dilatation of the superior mesenteric vein above a mesocaval shunt[17].

Duplex is less useful in the assessment of hepatic and splanchnic arterial flow[24]. The vessels are relatively short, tortuous and deeply situated, making them difficult to image and the hepatic arterial supply is frequently multiple. Post liver transplantation the anatomy may be even harder to demonstrate and scanning of the hepatic artery is currently too time consuming and inaccurate to make it clinically useful in detecting hepatic arterial thrombosis. Intra-arterial Doppler flow probes are now being developed and combined with angiography may ultimately provide the best way to quantitatively measure hepatic and splanchnic arterial flow[24].

In summary, Duplex scanning potentially provides a non-invasive way of assessing liver blood flow in many clinical situations including the assessment of portal vein patency, direction of flow, surgical porto-systemic shunting and liver transplantation. Its accuracy has been validated *in vitro* and in experimental animals[25,26], but problems do exist in using this technique in clinical practice (see appendix A). Improvements both in hardware and software as well as the development of colour flow mapping are likely to be reflected in a greater use of Duplex ultrasound in liver blood flow studies in the future. It offers one of the best approaches to the non-invasive assessment of portal flow but is not yet capable of reliably assessing hepatic arterial flow.

### *X-Ray angiography*

Angiography, or radiographic imaging of blood vessels, has a well established role in the diagnosis of liver disease and portal hypertension and is widely used in clinical practice for obtaining high quality vessel images[27]. As well as anatomical information, however, information on blood flow is also potentially available.

The techniques available for measuring flow using angiography are based on one of two principles. The first approach uses the principle that when an indicator is injected at constant rate into a blood vessel, the degree of dilution is proportional to blood flow, and the concentration in blood after mixing will be lower with higher flow and vice versa[28]. The main problem with this technique is accurate densitometric calibration. The second technique involves the measurement of the time taken for the passage of a bolus of contrast material between two sites but unfortunately precise timing of the passage of a dispersing bolus is often difficult to achieve[29]. In a new approach to this problem flow is determined by computer analysis of contrast concentration profiles as a function of time and distance along a vessel segment[29,30]. Another solution has been to assess relative flow by using two injections and measuring superior mesenteric, hepatic and splenic arterial flow relative to cardiac output[31,32].

The measurement of liver blood flow by angiographic techniques has largely been limited to hepatic arterial studies because catheter access to the portal system is not a routine procedure. Indirect portography, where contrast is injected into either the superior mesenteric artery or coeliac artery and imaged as it passes out into the portal system results in generally poor images unsuitable for flow analysis. Following direct insertion of a catheter into the portal system, Sovak *et al.*[33] used a computer to calculate the displacement of lipoidal droplets per frame. The average velocity ranged from 15.5 to 24.4 cm/sec in 6 normal patients and decreased during inspiration. Recently Iwanaga *et al.*[34] used this method[35] as a "gold standard" to test the validity of a Doppler duplex system for measuring portal blood flow in 10 patients with liver disease and found a significant correlation ($r = 0.970$) between the maximum portal blood flow velocity by duplex ultrasound and the mean velocity calculated from cineangiographic methods.

Although it requires vascular catheterisation X-ray angiography is still the modality of choice for critical morphological vascular studies. That X-ray angiography has not been widely used for measuring blood flow is due in part, we believe, to the use of inappropriate algorithms for processing the image data[29,36]. The method does, however, have great potential especially when combined with lower dose Digital Subtraction Angiography and new low osmolarity non-ionic contrast agents[37]. Minipuncture needles and catheters[38] have led to increased safety of the technique and the equipment and expertise is potentially available in many centres.

*Nuclear magnetic resonance*

Nuclear Magnetic Resonance (NMR) imaging is a noninvasive imaging modality that is rapidly gaining clinical acceptance, although widespread introduction has been delayed by expense. Flow detection with NMR spectroscopy has been explored for more than 30 years[39–41]. When NMR imaging was first performed in the late 1970s signal loss was noted within arteries and attributed to high flow rates[42]. In the early 1980s, several causes of increased signal intensity were described, generally associated with slow flow in veins and dural sinuses[43,44]. Understanding these flow phenomena has provided the basis for the development of specialized NMR imaging sequences intended to quantitate blood flow measurements. Several methods have been proposed to quantify blood flow[45–47] but at this stage their relative merits in terms of spatial and velocity resolution and image acquisition times have not been completely evaluated, nor has liver blood flow measurement been considered specifically. We suspect, however, that this will be an area of great development in the future.

*Dye Dilution Techniques*

*Plasma disappearance methods*

Attempts have been made since the middle of this century to measure liver blood flow by dye infusion methods[48]. Certain organic dyes are extracted by the hepatocytes and if the rate of extraction is measured, liver blood flow can be calculated using Fick's Principle[49]. The liver plasma flow (LPF) is defined as:

$$LPF = \Pi/[C_a(t)\text{-}C_v(t)]$$

where, $\Pi$ is rate of removal of the dye from the circulation by the liver in mg/min, $C_a(t)$ and $C_v(t)$ are the dye concentrations in mg/ml of the blood entering and leaving the liver respectively, leading to LPF measured in ml/min. LPF can be converted into liver blood flow if the value for the haematocrit is known.

The first dye used was bromsulphalein[50] but indocyanine green is now used more commonly as it is more specifically extracted by the liver[51]. The first measurements made using this substance employed the constant infusion method but Caesar *et al.*[51] have shown that analysis of plasma disappearance curves after a bolus injection gives nearly identical results. Hepatic extraction is usually measured by hepatic vein sampling, however, a method requiring only peripheral vein sampling and utilising pharmacokinetic modelling has been described and validated in normal subjects[52]. This method has, however, been criticised when applied to patients after liver transplantation[53] and it may be unreliable in patients with liver disease[54].

Pirttiaho *et al.*[55] estimated liver blood flow by fast intravenous injection of indocyanine green (0.5 mg/kg body weight). They found that the liver blood flow in 5 normal patients was $1258 \pm 119$ ml/min and that there was a close correlation ($r = 0.88$) with dynamic $^{99m}$Tc-sulphur colloid imaging but not with values obtained using the $^{133}$Xe clearance technique.

The advantage of dye clearance techniques is that they are relatively simple. Inaccuracies arise, however, when extrahepatic removal of the dye occurs[50] or when it is used in patients with liver disease[54]. These inaccuracies, combined with the development of other, more accurate methods, for measuring liver blood flow, have resulted in dye clearance methods being used less commonly. For many years, however, they were the best technique available and much pioneering work was done using them.

*Radioisotopic methods*

The concept of using radioactive tracers to help in the assessment of liver blood flow and perfusion is attractive. Three basic groups of techniques have been described.

### *A. Diffusible Gas Tracers*

Kety[56] introduced the principle of "local tissue clearance" or "washout" of rapidly diffusing isotopes as a way of measuring blood flow. Initially small amount of radioactive $^{24}$Na was used but later inert and lipid-soluble gases such as $^{85}$Kr and $^{133}$Xe[57] were found to be more valuable with the cellular membrane not constituting a barrier to diffusion[58].

Following injection of an arterial or portal venous bolus of gas dissolved in saline the elimination of these elements is in most situations only limited by the rate of capillary blood flow. Such an isotope will be eliminated in the form of a monoexponential function (giving a straight line when plotted on a logarithmic scale) if the tissue is uniformly perfused. Externally placed scintillation detectors are used to record the clearance curve. Fick's principle is then used in the analysis of the data and from a series of washout curves liver blood flow can be calculated.

### *B. Radio-labelled Colloids*

In this technique colloid-bound radionuclides are administered intravenously and the rate constant of liver uptake is measured either by multiple blood sampling or external scintillation counting. The Fick principle is then applied to calculate blood flow, with the assumption that extraction efficiency is 100%.

Various colloids have been used with different radionuclides. The first work was with $^{32}$P labelled chromic phosphate which is a pure $\beta$-particle emitter and cannot be detected by external counting[59,60]. It does, however, have a relatively high extraction efficiency at 95%. Colloidal $^{198}$Au has been used for external counting[61,62] but has a lower extraction efficiency (80% or less). Now $^{99m}$Tc-labelled sulphur colloid is most frequently used as it has a high extraction efficiency and can be counted externally. Dynamic images are acquired via a gamma camera and an on-line computer system and the assumption is made that the liver and spleen have an equal extraction efficiency for colloidal particles[63] and that this is close to 100%. Unfortunately, although these assumptions are probably valid in normal subjects, they may not be true in patients with liver disease[64]. Analysis of the time variation in liver activity is performed following bolus intravenous injection. The arterial and portal components are separated by their times of arrival at the liver. Several different methods have been described to estimate fractional hepatic arterial flow using hepatic artery, portal vein, and reference organ time activity curves[63–67].

### *C. Hepatosplenic Radionuclide Angiography*

This technique is based on the use of $^{99m}$Tc-pertechnetate which is not extracted by the liver. A bolus injection is given intravenously and dynamic images are acquired during its first pass phase[68]. The original technique[69] has been modified[70] and is now reported to be more reproducible[68]. Sarper *et al.*[68] generated first-pass radioactivity versus time curves by following a rapid intravenous injection of 740 MBq of $^{99m}$Tc-pertechnetate. For analysis, two time points were identified: the arrival time of activity in the liver, $t_o$, and the time of maximum activity of the abdominal organs, $t_c$. The former was estimated from the liver time-activity curve; the latter from the kidney time-activity curve and the accuracy of the method depends on there being normal perfusion of the kidneys which act as a reference organ. The gradients of the liver curve from $t_o$ + 7 seconds and from $t_c$ to $t_c$ + 7 seconds, calculated by linear least-squares regression analysis, are $G_o$ and $G_c$ respectively. A hepatic perfusion index (HPI) is then defined as:

$$HPI = G_c/(G_o + G_c).$$

This method was applied to 7 normal volunteers and 57 patients with biopsy proven cirrhosis. The HPI was 66 ± 7% for the normals and ranged from 8–59 for patients with liver cirrhosis[68].

### *Advantages and limitations of radioisotope methods*

The use of radio-labelled diffusible gas tracers has gained acceptance and popularity for measuring cerebral blood flow, where an inhalation technique can be used successfully. Unfortunately the technique is not as accurate for measuring liver blood flow and the washout curves are frequently not monoexponential[71], perhaps due to recirculation of the tracer, the fact that liver tissue may not be homogenously perfused or because there is incomplete clearance of tracer during its first passage[72]. In addition, the need to catheterise either the hepatic portal or arterial system is a major disadvantage and one of the reasons why the technique has failed to gain widespred popularity in hepatic studies.

Colloid bound tracers and radionuclide angiography can not provide absolute values for flow but they can provide valuable information about the relative contribution of the hepatic arterial and portal systems. Such an index may be of more interest than absolute flow values in certain disease states such as cirrhosis. However, the background scatter of tracer and the affinity to fat which many tracers have, makes the measurements difficult to evaluate. Colloids also have a range of particle sizes and hence a range of values of extraction efficiency.

### METHODS MEASURING TISSUE PERFUSION

### *Radioactive Microspheres*

If a bolus of tracer is well mixed in the afferent blood supplying an organ, then it will be distributed to different parts of the organ in exactly the same way as the blood which is transporting it. This is called the indicator fractionation principle. This principle has been used to quantify regional blood flow distribution using radio-labelled particles, diffusible indicators and autoradiography[73,74]. Microspheres are chosen to be of a size (10 – 15$\mu$m) which will just lodge in the capillary circulation. The injection can be given some time before local distribution of the trapped spheres is measured, a procedure usually carried out post-mortem by taking biopsies of the tissue being studied and measuring radioactivity in a well counter.

Using this technique Greenway *et al.*[75] studied the regional distribution of portal and hepatic blood flow in the liver by injecting $^{14}C$ amd $^{51}Cr$-microspheres into the portal vein and hepatic artery of 12 cats and 15 dogs. They found the liver homogeneously perfused from both systems in contrast to other work using different techniques[76,77]. The technique has also been used to study the vascularity of experimental liver tumours and in particular the relative role of portal and arterial blood supply to these tumours[78-81].

The microsphere method is useful for providing values of blood flow in animal studies where sacrifice of the animal occurs. Impaction of microspheres must, however, affect local flow and with multiple injections before sacrifice this may become a significant source of artefact. To minimize this problem, injection quantities are made as small as possible, but this militates against accurate measurement of regional flow, especially in regions with low volume flow. Its major disadvantage is, however, that it cannot be used clinically.

### *Heat Exchange Methods*

The concept of measuring tissue blood flow using heat clearance techniques was first suggested by Gibbs[82]. This method requires a heated thermocouple, maintained at a certain temperature (2–4°C) above that of the surrounding tissue, to be either placed onto the liver surface or inserted into the liver tissue. The temperature of the needle is dependent on local blood flow – increased perfusion tends to cool the needle, whereas reduced perfusion allows it to heat up. Measurement of the energy required to maintain the temperature increment constant therefore can be regarded as giving an indirect measurement of flow[82-85]. However, values will depend on the exact position of the probe and the metabolic state of the liver[86]. The method is, therefore, only a semiquantitative approach to flow and because of its invasiveness has not yet found widespread favour for liver studies.

### *Hydrogen Electrode*

This method was introduced by Auckland and Bower[87] and further developed by Fieschi *et al.*[88,89] and by Bozzao *et al.*[90]. Molecular hydrogen is administered with the respiration gas until the tissue reaches saturation. The hydrogen supply is then turned off and its clearance rate is determined polarographically through platinum electrodes placed on, or into, the liver. A current is generated at the electrode surface by oxidation of molecular hydrogen to hydrogen ions. This current declines as hydrogen is removed and the steepness of the clearance curve correlates directly with the magnitude of the total liver blood flow and reflects perfusion within a radius of approximately 5 mm of the electrode. The calculation of tissue blood flow from hydrogen clearance curves is based on the theory developed by Kety,[91] and the method has been reviewed and simplified by Young[92].

Gouma *et al.*[77], using a hydrogen electrode applied to the surface of porcine liver, found that calculated liver blood flow measurements using this method gave much lower values than those obtained using the indocyanine green clearance method and in addition flow fell by over 90% if the hepatic artery was ligated. They concluded from these experiments that the surface of the liver is mainly supplied from the arterial system. Nishiwaki *et al.*[93] used the hydrogen clearance method and transit-time ultrasonic blood flowmetry to investigate blood flow after liver transplantation in 40 mongrel dogs and found reductions in both hepatic arterial and portal venous flow after transplantation.

The advantages of this method are that it can provide unlimited measurements of liver blood flow without significant alteration in physiological variables. There is no evidence that the administration of the hydrogen itself significantly alters flow. Despite this, most investigators have not used the technique, mainly due to concern over the inflammability and explosiveness of pure hydrogen gas. In addition the method is not continuous, cannot handle rapit changes of flow and may reflect arterial rather than venous inflow[77]. It may also be inaccurate if the liver is not homogenously perfused.

*Oxygen Electrode*

An oxygen electrode consists of a noble metal cathode maintained at a negative potential with respect to a reference electrode. It is placed on the surface of the organ to be studied and oxygen diffusing from the tissue to the cathode surface is reduced when the potential is applied, giving rise to a current[94]. A naked electrode is subject to "poisoning" by electrophoretic deposition of tissue protein on its surface but this can be prevented by covering the electrode with a gas-permeable membrane[95]. When the oxygen consumption of the electrode is low, tissue oxygen is not disturbed and so a direct measurement of the partial pressure ($pO_2$) is obtained[96]. However, if the oxygen consumption of the electrode is high the electrode will measure the rate of supply of oxygen to the tissue and this is dependent on local blood flow[97,98].

Ji *et al.*[99] applied a microneedle electrode to rat liver and found that tissue $pO_2$ values were different at periportal and perihepatic sites. Kram and Shoemaker[100] applied a "miniature" oxygen electrode in a single illustrative case to human cirrhotic liver to measure tissue $pO_2$ and their instrument responded to both changes in local organ blood flow and arterial $pO_2$. In these two studies electrode readings were not related to portal venous flow. We compared readings from a membrane covered (Clark type) flow dependent oxygen electrode applied to the surface of rabbit liver, with those from an electromagnetic flowmeter on the portal vein. We found that under operative conditions, changes in oxygen electrode readings correlated well with portal blood flow as measured by an electromagnetic flowmeter over a range of flow rates[101].

This technique can give a continuous and instantaneous measurement of portal venous inflow when hepatic arterial inflow is undisturbed. However, it is invasive as the electrode must be applied directly onto the liver surface. In addition it only gives a measure of flow in the tissue immediately below the electrode and no absolute value for flow can be calculated. Potentially, however, it can be used on human liver either at laparotomy or laparoscopy.

### *Laser Doppler*

Laser Doppler is a relatively new technique (1972)[102] for measuring local blood flow. The device consists of a helium-neon laser and an optic fibre which transmits this light to the surface of the tissue to be studied. Light that is scattered by red blood cells undergoes a frequency shift and a portion of this spectrally-broadened light is transmitted back by a fibre light-guide to two photodetectors. This signal is analyzed and the relative portion of light which has undergone Doppler shift is proportional to velocity of blood flow. The microvascular bed consists of an intricate network of small blood vessels and hence the angle between the red cell velocity vectors and the beam propagation vectors of the scattered light can be regarded as random[103].

This technique gives a continuous measure of red cell motion in the outermost layer of the tissue under study with little or no influence on blood flow. The depth to which the beam penetrates varies with the tissue being studied[103,104,76] and flow is likely to be measured in a volume of approximately 0.6–1.3 $mm^3$ when the probe is applied to the liver surface.

Laser Doppler has been studied *in vitro* by measurement of liquid flow through small-bore tubes and a coefficient of variation for readings of 6%[105] confirmed its accuracy. *In vivo* it has been used extensively to measure skin blood flow[106] but less has been written on its use and limitations for estimating liver blood flow[76,107].

Arvidsson *et al.*[76], using Laser Doppler flowmetry in pigs, investigated liver blood flow and confirmed previous work[77] with hydrogen clearance methods that the liver surface is mainly supplied from the arterial system. Laser doppler has also been used to study blood flow to experimental liver metastases[108].

The advantages of the method are that it can provide an instantaneous and continuous measurement of microcirculatory flow in a way that does not alter flow. Its disadvantages are that the probe must be applied directly onto the liver, flow can not be measured in absolute units, the absolute volume of tissue measured is not known and only surface flow is assessed (needle probes have not been used in the liver as haematoma formation around the probe tip would make readings unreliable).

### CONCLUSION

Currently the measurement of hepatic blood flow and perfusion is fraught with difficulties. There are often large variations in both flow and perfusion measurements, not only between techniques but also between different groups using the same technique. Some of these differences may be due to the methods and conditions used and others are undoubtedly caused by complexities of the liver circulation which we do not yet understand.

Most importantly, we are still looking for a reliable method of non-invasively assessing liver blood flow in the clinical context. Duplex Doppler Ultrasound offers a good way of assessing portal vein flow but its many inaccuracies should be borne in mind. Clinical measurement of hepatic and splanchnic arterial flow is more difficult and duplex does not yet have the accuracy to perform this function reliably. Intraarterial Doppler flow probes, flow analysis of digital x-ray angiograms and NMR may have a role in the future. Radiolabelled colloids or hepatosplenic radionuclide angiography can provide valuable information about the relative contribution of the portal and arterial system but are still largely research tools and are less accurate in the presence of pathology. Currently, investigators are best advised to familiarise themselves with a range of techniques as it is apparent that no single method is able to fulfil all the requirements of either basic research or routine clinical practice.

### APPENDIX A

### DOPPLER'S LIMITATIONS AND SOURCES OF ERRORS

Although duplex instrumentation is now widely accepted as valid for most vascular applications, there are several disadvantages which have prevented total acceptance of the method[109]. These have been well documented and summarised by Burns[16] and Merritt[110]. Briefly, they are as follows:

(a) When used to measure laminar flow in a vessel and the beam width is less than the diameter of the vessel, only the central portion of the vessel lumen will be insonated[111] leading to an error in estimating velocity and hence flow.

(b) Errors can arise in estimating blood vessel diameter due to: (1) Imaging not being perpendicular to the longitudinal axis of the vessel. (2) Poor resolution of the imaging transducer. (3) Pulsatility of blood flow, causing variation in vessel diameter with time. (4) Observer variability[112]. (5) Only a short length of vessel being available for imaging such as occurs with the portal vein[13,25].

(c) Sampling problems: Nearly all current pulse mode Doppler machines are based on centre-line measurements of peak velocity and spectral broadening[113]. Abnormalities may be missed due to failure to sample nearer the wall where flow disturbances are most likely to occur[114,115].

(d) Errors due to beam angle: the magnitude of velocity is the function of the cosine of the intercept angle between beam direction and the blood vessel. The flow-volume rate calculation equation is a trigonometrical function of the angle between the beam direction and the blood flow. Errors vary considerably with that angle, being minimal in the angle range 55–76°[116].

*Acknowledgements*

We thank Dr. S. Padayachee, Division of Radiological Sciences, Guy's Hospital School of Medicine, London, for valuable discussion on application of Doppler ultrasound to measure liver blood flow and also we thank Dr. C. Piasecki for valuable discussions on application of oxygen electrode to measure liver blood flow. D.J.H. is grateful for the support of the Leverhulme Trust.

*References*

1. Richardson, P.D.I. and Withrington, P.G. (1981) Liver blood flow I.Intrinsic and nervous control of liver blood flow. *Gastroenterology*, **81**, 159–173
2. Richardson, P.D.I. and Withrington, P.G. (1981) Liver blood flow: II. Effects of drugs and hormones on liver blood flow. *Gastroenterology*, **81**, 356–375
3. Fabre, P. (1932) Utilisation des forces electromotrices d'enrigistrement des variations de vitesse des liquids conducteurs: un nouvel hemodromograph sans palette dans le sang. *C. R. Acad. Sci. Paris*, **194**, 1097–1098
4. Webster, J.G. (1978) Measurement of flow and volume of blood. In: Medical instrumentation: Application and Design. Ed. Webster J G. Boston: Houghton Mifflin, ch. 8
5. Harper, A.M., Lorimer, A.R. and Thomas, D.L. (1974) Methods of measuring blood flow. In: Scientific Foundation of Anaesthesia, Ed. Scurr C, Feldman, S. London: Heinemann, pp. 28–44
6. Khouri, E.M. and Gregg, D.E. (1963) Miniature electromagnetic flow meter applicable to coronary arteries. *J. Appl. Physiol.*, **18**, 224
7. Wyatt, D.G. (1984) Blood flow and blood velocity measurement in vivo by electromagnetic induction. *Med. Biol. Eng. Comput.*, **22**, 193–211
8. Meisner, H. and Messmer, K. (1970) Significance and limitations of electromagnetic blood flowmetry, Experimental and Clinical results. *Progr. Surg.*, **8**, 124–144
9. Drapanas, T., Kluge, D.N. and Schenk, W.G. (1960) Measurement of hepatic blood flow by bromsulphalein and by the electromagnetic flowmeter. *Surgery*, **48**, 1017–1021
10. Price, J.B., Britton, R.C., Peterson, L.M., Reilly, J.W. and Voorhees, A.R. (1965) The validity of chronic hepatic blood flow measurements obtained by the electromagnetic flow meter. *J. Surg. Res.*, **V(7)**, 313–317
11. Bradley, S.E., Inglefinger, F.J., Bradley, G.P. and Curry, J.J. (1945) The estimation of hepatic blood flow in man. *J. Clin.Invest.*, **24**, 890
12. Satomura, S. (1959) Study of the flow pattern in peripheral arteries by ultrasonics. *Nihon Onkyo-gakkai, Shi*, **15**, 151
13. Taylor, K.J.W. and Burns, P.N. (1985) Duplex Doppler scanning in the pelvis and abdomen. *Ultrasound Med. Biol*, **11**, 643–658
14. Okazaki, K., Miyazki, S. and Onishi, S. (1987) Noninvasive measurements of portal blood flow. *Gastroenterology*, **93**, 656–657
15. Baker, D.W. (1970) Pulsed ultrasonic Doppler blood-flow sensing. *IEEE Trans. Sonics Ultrasonic*, **17(3)**, 170
16. Burns, P.N. (1987) The physical principles of doppler and spectral analysis. *J. Clin. Ultrasound*, **15**, 567–590
17. Koslin, D.B. and Berland, L.L. (1987) Duplex Doppler examination of the liver and portal venous system. *J. Clin. Ultrasound*, **15**, 675–686
18. Ohnishe, K., Saito, M., Koen, H., Nakayama, T., Normura, F. and Okuda, K. (1985) Pulsed Doppler flow as a criterion of portal venous velocity : Comparison with cineangiographic measurements. *Radiology*, **154**, 495–498
19. Sovak, M., Soulen, R.L. and Reichle, F.A. (1971) Blood flow in the human portal vein. A cineradiographic method using particulate contrast medium. *Radiology*, **99**, 531–536
20. Ackroyd, N., Lane, R. and Dart, L., *et al.* (1984) Colour-coded carotid Doppler imaging: An angiographic comparison of 324 bifurcation. *Aust. NZ. J. Surg.*, **54**, 509–517
21. Okazaki, K., Miyazaki, M., Onishi, S. and Ito, K. (1986) Effect of food intake and various extrinsic hormones on portal blood flow in patients with liver cirrhosis demonstrated by pulsed doppler with the octoson. *Scand. J. Gastroenterol.*, **21**, 1029–1038
22. Moriyasu, F., Nishida, O. and Ban, N., *et al.* (1986) Congestion index off the portal vein. *AJR*, **146**, 735–739

23. Nelson, R.C., Lovett, K.E., Chezmar, J.L., Moyers, J.H., Torres, W.E., Murphy, F.B. and Bermardino, M.E. (1987) Comparison of pulse Doppler sonography and angiography in patients with portal hypertension. *AJR*, **149**, 77–81
24. Barbara, L., Bolondi, L. and Bosch, J., *et al.* (1990) The value of Doppler US in the study of hepatic hemodynamics. *J. Hepatol.*, **10**, 353–355
25. Miyatake, K., Okamoto, M., Kinoshita, N., Izumi, S., Owa, M., Takao, S., Sakakibara, H. and Nimura, Y. (1984) Clinical applications of a new type of real-time two-dimensional doppler flow imaging system. *Am. J. Cardiol.*, **54**, 857–868
26. Seifalian, A.M., Young, J.M. and Hobbs, K.E.F. (1988) Quantitative assessment of portal vein blood flow by duplex ultrasound scanning. *Clin. Radiol.*, **39**, 681
27. Dick, R. (1983) Portal and hepatic venous system. In: Techniques in diagnostic radiology. Eds, G.H.Whitehouse and B.S.Worthington. Blackwell Scientific Publications, 165–182
28. Hillal, S.K. (1966) The determination of the blood flow by a radiographic technique. *Am. J. Roentgenol.*, **96**, 896–906
29. Seifalian, A.M., Hawkes, D.J., Colchester, A.C.F. and Hobbs, K.E.F. (1989) A new algorithm for deriving pulsatile blood flow waveforms tested using simulated dynamic angiographic data. *Neuroradiology*, **31**, 263–269
30. Colchester, A.C.F. (1985) The effect of changing PaCo2 on cerebral artery calibre estimated by a new technique of dynamic quantitative digital angiography. Phd Thesis, University of London
31. Lantz, B.M.T., Link, D.P., Foerster, J.M. and Holcroft, J.W. (1980) Angiographic determination of splanchnic blood flow. *Acta Radiologica Diagnosis*, **21**, 3–10
32. Kedem, D., Kedem, D., Smith, D.W., Dean, R.H. and Brill, A.B. (1978) Veocity distribution and blood flow measurements using videodensitometric methods. *Invest. Radiol.*, **13(1)**, 46–56
33. Sovak, M., Soulen, R.L. and Reichle, F.A. (1971) Blood flow in the human portal vein. A cineradiographic method using particulate contrast medium.*Radiology*, **99**, 531–536
34. Iwanaga, T., Koyannagi, N. and Sugimachi, K. (1987) Validity of ultrasonic duplex system for measurement of the portal blood flow in patients with liver disease. *Japanese J. Surgery*, **17(1)**, 58–59
35. Reichle, F.A., Sovak, M., Soulen, R.L. and Rosemond, G.P. (1972) Portal vein blood flow determination in the unanesthetized human by umbilicoportal cannulation. *J. Surg. Res.*, **12**, 146–150
36. Du Boulay, G.H., Brunt, J., Colchester, A., Hawkes, D., Wallis, A. and Wicks, D. (1987) Volume flow measurement of pulsatile flow by digitised cine angiography. *Acta Radiologica*, **Suppl(13)**, 59–62
37. Bettman, M.A. and Morris, T.W. (1986) Recent advances in contrast agents. *RCNA*, **24(3)**, 347–357
38. Cope, C. (1986) Minipuncture angiography. *RCNA*, **24(3)**, 359–367
39. Carr, H.Y. and Purcell, E.M. (1950). *Phys. Rev.*, **94**, 630
40. Garroway, A.N. (1974) Velocity measurements in flowing fluids by NMR. *J.Phys. D: Appl. Phys.*, **7**, 159–163
41. Hahn, E.L. (1960) Detection of sea water motion by nuclear procession. *J. Geophys. Res.*, **65**, 776–777
42. Hawkes, R.C., Holland, G.N., Moore, W.S. and Worthington, B.S. (1980) Nuclear magnetic resonance (NMR) tomography of the brain: a preliminary clinical assessment with demonstration of pathology. *J.Comput. Assist. Tomogr.*, **4**, 577–586
43. Waluch, V. and Bradley, W.G. (1984) NMR even echo rephasing in slow laminar flow. *J. Comput. Assist. Tomogr.*, **4**, 594–598
44. Bradley, W.G. and Waluch, V. (1985) Blood flow: Magnetic resonance imaging. *Radiology*, **154**, 443–450
45. Ridgway, J.P., Turnbull, L.W. and Smith, M.A. (1987) Demonstration of pulsatile cerebrospinal-fluid flow using magnetic resonance phase imaging. *Bri. J. Radiology*, **60**, 423–427
46. Shimizu, K., Matsuda, T., Sakurai, T., Fujita, A., Ohara, H., Okamura, S., Hashimoto, S., Mano, H., Kawai, C. and Kiri, M. (1986) Visualization of moving fluid: Quantitative analysis of blood flow velocity using mr imaging. *Radiology*, **159**, 195–199
47. West, D.J., Tarnawski, M., Graves, M.J., Taylor, M.G., Padayachee, T.S., Ayton, V.T. and Smith, M.A. (1988) Blood flow imaging by magnetic resonance. *Medicamundi*, **33(3)**, 101–111
48. Bradley, S.E. and Curry, J.J. (1945) The estimation of hepatic blood flow in man. *J. Clin. Invest.*, **24**, 890–987
49. Fick, A. (1870) Uber die Messung des Blutquantums in den Herzventrikeln. *S. B. Phys-med. Ges. Wurzb.*, **21**, 171–180

50. Brauer, R.W., Pessotti, R.L. and Krebs, J.S. (1955) The distribution and excretion of 35S-labeled Sulfobromophthalein-sodium administered to dogs by continuous infusion. *J. Clin. Invest.*, **34**, 35–40
51. Caesar, J., Shaldon, S., Chiandussi, L. and Sherlock, S. (1961) The use of indocyanine green in the measurement of hepatic blood flow and as a test of hepatic function. *Clin. Sci.*, **21**, 43–57
52. Grainger, S.L., Keeling, P.W.N., Brown, I.M.H., Marigold, J.H. and Thompson, R.P.H. (1983) Clearance and non-invasive determination of the hepatic extraction of indocyanine green baboons and men. *Clin. Sci.*, **64**, 307–412
53. Clements, D., West, R. and Elias, E. (1986) Comparison of two methods of estimating liver blood flow in patients with liver disease using indocyanine green. *Gut*, **27**, A613-4
54. Combes, B. (1960) Estimation of hepatic blood flow in man and dogs by $^{131}$I-labelled Rose Benfal:Simultaneous comparison with sulfabromophthalein sodium. *J. Lab. Clin. Med.*, **56**, 537–543
55. Pirttiaho, H., Pitkanen, U., Rajasalmi, M. and Ahonen, A. (1980) Comparison of three methods of measuring liver blood flow. *Acta Radiological Diagnosis*, **21**, 535-539.
56. Kety, S.S. (1949) Measurement of regional circulation by the local clearance of radioactive sodium. *Am. Heart. J.*, **38**, 321--328
57. Lassen, N.A. and Munck. (1955) Cerebral blood flow in man determined by the use of radioactive Krypton. *Acta. Physiol. Scand.*, **33**, 30–49
58. Lassen, N.A. (1964) Muscle blood flow in normal man and in patients with intermittent claudication evaluated by simultaneous $^{133}$Xe and $^{24}$Na clearances. *J. Clin. Invest.*, **43**, 1805–1812
59. Dobson, E.L. and Jones, H.B. (1952) The behaviour of intravenously injected particulate material. Its rate of disappearance from the blood stream as a measure of liver blood flow. *Acta. Med. Scand.*. **144**, 71–85
60. Dobson, E.L., Warner, G.F., Finney, C.R. and Johnston, M.E. (1953) The measurement of liver circulation by means of the colloid disappearance rate. I. Liver blood flow in normal young men. *Circulation*, **7**, 690–695
61. Riddell, A.G., Griffiths, D.B., McAlister, J.M. and Osborn, S.B. (1957) The measurement of liver blood flow with colloidal radiogold ($^{198}$Au). *Clin. Sci.*, **16**, 315–324
62. Fauvert, R., Benhamou, J., Nicollo, S. and Loverdo, A. (1958) La clearance de l'or colloidal radio-actif (198-Au). I. Valeurs normales et valeurs pathologiques. *Rev. Franc. Etud. Clin. Biol.*, **3**, 762–766
63. Fleming, J.S., Humphries, N.L.M., Karran, S.J., Goddard, B.A. and Ackery, D.M. (1981) In vivo assessment of hepatic-arterial and portal-venous components of liver perfusion:Concise communication. *J. Nucl. Med.*, **22(1)**, 18–21
64. Wright, E.P., Barber, R.W. and Ritson, A. (1982) Relative hepatic arterial and portal flow in liver scintigraphy. *Nucl. Med. Commun.*, **3**, 273–279
65. Fleming, J.S., Ackery, D.M., Walmsley, B.H. and Karran, S.J. (1983) Scintigraphic estimation of arterial and portal blood supplies to the liver. *J. Nucl. Med.*, **24**, 1108–1113
66. Parkin, A.C., Robinson, P.J., Baxter, P., Leveson, S.H., Wiggin, P.A. and Giles, G.R. (1983) Liver perfusion scintigraphy – Method, Normal range and laparotomy correlation in 100 patients. *Nucl. Med. Commun.*, **4**, 395–402
67. Britten, A.J., Fleming, J.S., Flowerdew, A.D.S., Hunt, T.M., Taylor, I., Karran, S.J. and Ackery, D.M. (1990) A comparison of three indices of relative hepatic perfusion derived from dynamic liver scintigraphy. *Clin.Phys. Physiol. Meas.*, **11**, 45–51
68. Sarper,R., Fajman, W.A., Rypins, E.B., Henderson, J.M., Tarcan, T.A., Galambos, J.T. and Warren, W.D. (1981) A noninvasive method for measuring portal venous/total hepatic blood flow by hepatosplenic radionuclide angiography. *Radiology*, **141**, 179–184
69. Biersack, H.J., Thelen, M. and Schulz, D. (1977) Die sequentielle hepatospleno szintigraphie zur quantitiven beurteilung der leberdurchblutung. *Fortschre, Roentgenstr*, **126**, 47–52
70. Rypins, E.B., Fajman, W. and Sarper, R. (1981) Radionuclide angiography of the liver and spleen. A non-invasive method of assessing portal venous/total hepatic blood flow ratio and portasystemic shunt patency. *Am. J. Surg.*, **142**, 574–579
71. Birtch, A.G., Casey, B.H. and Zakheim, R.M. (1967) Hepatic blood flow measured by the $^{85}$Kr clearance technique. *Surgery*, **62**, 174–180
72. Mackenzie, R.J., Leiberman, D.P., Mathie, R.T. (1976) Liver blood flow measurement. The interpretation of $^{133}$Xe clearance curves. *Acta Chir.Scand*, **142**, 519–525
73. Heymann, M.A., Payne, B.D., Hoffman, J.I.E. and Rudolph, A.M. (1977) Blood flow measurements with radionuclide labelled particles. *Prog. Cardivas. Dis.*, **20**, 55–79

74. Romeo, J.M., Lopez-Farre, A., Martin-Paredero, V. and Lopez-Novoa, J.M. (1990) Hepatic haemodynamic changes after portacaval anastomosis in normal, cirrhotic and chronic prehepatic portally hypertensive rats. *Br. J. Surg.*, **77**, 335–338
75. Greenway, C.V. and Oshiro, G. (1972) Intrahepatic distribution of portal and hepatic arterial blood flows in anaesthetized cats and dogs and the effects of portal occlusion, raised venous pressure and histamine. *J. Physiol.*, **227**, 473–485
76. Arvidsson, D., Svensson, H. and Haglung, U. (1988) Laser Doppler flowmetry for estimating liver blood flow. *Am. J. Physiol.*, **254**, G471-6
77. Gouma, D.J., Coelho, J.C., Schlegel, J., Fisher, J.D., Li, Y.G. and Moody, F.G. (1986) Estimation of hepatic blood flow by hydrogen gas clearance. *Surgery*, **99(4)**, 439–444
78. Ackerman, N.B., Lien, W.N., Kondi, E.S. and Silverman, N.A. (1969) The blood supply of experimental liver metastases. *Surgery*, **66**, 1067–1072
79. Archer, S.G., Gray, B.N. (1989) Vascularization of small liver metastases. *Br. J. Surg.*, **76**, 545–548
80. Nott, D.M., Grime, S.J., Yates, J., Day, D.W., Baxter, J.N., Jenkins and Cooke, T.G. (1989) Changes in the hepatic perfusion index during the development of experimental hepatic tumours. *Br. J. Surg.*, **76**, 259–263
81. Romeo, J.M., Lopez-Farre, A., Martin-Paradero, V. and Lopez-Novoa. (1990) Hepatic haemodynamic changes after portacaval anastomosis in normal, cirrhotic and chronic prehepatic portally hypertensive rats. *Br. J. Surg.*, **77**, 335–338
82. Gibbs, F.A. (1933) A thermoelectric blood flow recorder in form of a needle. *Proc. Soc. Exp. Biol. Med.*, **31**, 141–146
83. Grabner, G. and Neumayer (1958) A continuous recording method for the estimation of liver blood flow in man. In: Proceedings Harey Tercentenary Congress. Ed. J. McMichael. pp386–392
84. Grayson, J. and Kinnear, T. (1962) Observations on temperature, blood flow and heat production in the human liver in relation to environment and to glucose and insulin administration. *Clin. Sci.*, **22**, 125–140
85. Newman, W.H., Curley, M.G., Summit, S.C., Bowman, H.F., DelHomme, G. and Dittmar, A. (1990) Simultaneous, continuous measurements of tissue perfusion and oxygenation. *Annual International Conf.IEEE Eng. Med. Biol. Soci.*,**12(3)**, 1036–1037
86. Grayson, J. and Johnson, D.H. (1953) The effect of adrenalin and noradrealin of the liver blood flow. *J. Physiol.*, **120**, 73--94
87. Aukland, K., Bower, B.F. and Berliner, R.W. (1964) Measurement of local blood flow with hydrogen gas. *Circulation Research*, **XIV**, 164–187
88. Fieschi, C., Bozzao, L. and Agnoli, A. (1965) Regional clearance of hydrogen as a measure of cerebral blood flow. *Acta Neurol. Scand*, **14(Supl)**, 46–52
89. Fieschi, C., Bozzao, L., Agnoli, A., Nardini, M. and Bartolini, A. (1969) The hydrogen method of measuring local blood flow in subcortical structures of the brain: Including a comparative study with the 14-C antipyrine method. *Exp. Brain Res.*, **7**, 111–119
90. Bozzao, L., Agnoli, A., Bartolini, A. and Fieschi, C. (1969) Local cerebral blood flow measured by clearance curves of hydrogen gas. In: Research of the cerebral circulation. Eds, J.S. Meyer, H. Lechner and O. Eichorn. Third International Salzburg Conference, 86-96, Ch C. Thomas Springfield/I11., USA
91. Kety, S.S. (1951) The theory and applications of the exchange of inert gas at the lungs and tissue. *Pharmacol. Rev.*, **3**, 1–41
92. Young, W. (1980) $^2$H-clearance measurement of blood flow: A review of techniques and polarographic principles. *Stroke*, **11**, 552–564
93. Nishiwaki, H., Ohira, M., Boku, T., Ishikawa, T., Nakagawa, H., Yata, K. and Umeyama, K. (1989) The measurement of hepatic circulation before and after orthotopic liver transplantation in dog by using transit time blood flow meter and H-2 clearance method. *Nippon-Geka-Gekkai-Zasshi*, **90(2)**, 243–9
94. Baumberger, J.P. and Goodfriend, R.B. (1951) Determination of arterial oxygen tension in man by equilibration through intact skin. *Federation Proc.*, **10**, 10–11
95. Clark, L.C. (1956) Monitor and control of blood and tissue oxygen tension. *Tran. Am. Soc. Artif. Intern. Organ.*, **2**, 41–48
96. Hunt, T.K., Rabkin, J., Jensen, J.A., Jonsson, K., Smitten, K.V. and Goodson, W.H. (1987) Tissue oximetry: An interim report. *World J. Surg.*, **11**, 126–132
97. Piasecki, C. (1985) First experimental results with the oxygen electrode as a local blood flow sensor in canine colon. *Br. J. Surg.*, **72**, 452–453

98. Piasecki, C. (1981) A new method for the assessment of gut viability. *Br. J. Surg.*, **68**, 319–322
99. Ji, S., Lemasters, J.J., Christenson, V. and Thurman, R.G. (1983) Selective increase in pericentral oxygen gradient in perfused rat liver following ethanol treatment. *Pharmacology Biochemistry and Behaviour*, **19**, 439–442
100. Kram, H.B. and Shoemaker, W.C. (1984) Method for intraoperative assessment of organ perfusion and viability using a miniature oxygen sensor. *Am. J. Surg.*, **148**, 404–7
101. Piasecki, C. and Seifalian, A.M. (1990) Continuous intraoperative monitoring of hepatic blood perfusion using a noninvasive surface electrode. *Dig. Dis. Sci.*, **35(3)**, 399–405
102. Riva, C., Ross, B. and Benedek, G. (1972) Laser Doppler measurements of blood flow in capillary tubes and retinal arteries. *Invest. Opthalmol.*, **11**, 936–944
103. Nilson, G.E., Tenland, T. and Obser, P.A. (1980) Evaluating of a laser Doppler flowmeter for measurement of tissue blood flow. *IEEE Trans. Biomed. Eng*, **27**, 597–604
104. Bonner, R. and Nossal, R. (1981) Model for laser Doppler measurements of blood flow in tissue.*Applied Optics*, **20**, 2097--2107
105. Damber, J.E., Lindahl, O., Selstan, G. and Tenland, T. (1982) Testicular blood flow measured with a laser Doppler flowmeter: acute effects of catecholamines. *Acta Physiol.Scand*, **115(2)**, 209–15
106. Swain, I.D. and Grant, L.J. (1989) Methods of measuring skin blood flow. *Phys. Med. Biol.*, **34**, 151–175
107. Shepheard, A.P., Riedel, G.L. and Ward, W.F. (1983) Laser Doppler measurements of blood flow within the intestinal wall and on the surface of the liver. In: Microcirculation of the alimentary tract, Ed. by A.Koo, R.F.Lam, L.M.Swaje. Singapore: World Scientific, pp115–129
108. Bloom, N.D., Kroop, E., Sadjadi, M., Jacobs, R., Ramaswamy, G. and Akerman, N.B. (1987) Enhancement of tumour blood flow and tumoricidal effect of Doxorubicin by intraportal epinephrine in experimental liver metastases. *Arch. Surg.*, **122**, 1269–1272
109. Jaffe, C.C. (1984) Doppler applications and limits of the method. *Clin. Diag. Ultrasound*, **13**, 1–10
110. Merritt, C.R.B. (1987) Doppler color flow imaging. *J. Clin. Ultrasound*, **15**, 591–597
111. Evans, D.H. (1982) Some aspects of the relationship between instantaneous volumetric blood flow and continuous wave Doppler ultrasound recording - 1. *Ultrasound Med.Biol.*, **8**, 606–609
112. Zoli, M., Marchesini, G., Brunori, A., Cordiani, M.R. and Pisi, E. (1986) Portal venous flow in response to acute beta blocker and vasodilatatory treatment in patients with liver cirrhosis. *Hepatology*, **6**, 1248–1251
113. Langlois, Y.E., Greene, F.M., Roederer, G.O., Jager, K.A., Phillips, D.J., Beach, K.W. and Strandness, D.E. (1984) Computer based pattern recognition of carotid artery Doppler signals for disease classification: Prospective validation. *Ultrasound Med.Biol.*, **10**, 581–595
114. Switzer, D.F. and Nanda, N.C. (1985) Doppler color flow mapping. *Ultrasound Med.Biol.*, **11**, 403–416
115. Rittgers, S.E. and Fei, D. (1988) Flow dynamics in a stenosed carotid bifurcation model - part II: Derived indices. *Ultrasound Med. Biol.*, **14**, 33–44
116. Stacey-Clear, A., Fish, P.J. (1984) Repeatibility of blood flow measurement using multichannel pulsed Doppler ultrasound. *Bri. J. Radiol.*, **57**, 419–420

*(Accepted by S. Bengmark 7 February 1991)*

